# Textural properties and adsorption behavior of Zn–Mg–Al layered double hydroxide upon crystal violet dye removal as a low cost, effective, and recyclable adsorbent

**DOI:** 10.1038/s41598-023-33142-x

**Published:** 2023-04-20

**Authors:** E. E. Abdel-Hady, Hamdy F. M. Mohamed, Sarah H. M. Hafez, Abdalla M. M. Fahmy, Abdelhamed Magdy, Aya S. Mohamed, Eman O. Ali, Hager R. Abdelhamed, Osama M. Mahmoud

**Affiliations:** Physics Department, Faculty of Science, Minia University, P.O. Box 61519, Minia, Egypt

**Keywords:** Materials science, Nanoscience and technology

## Abstract

The preparation of adsorbents plays a vital role in the adsorption method. In particular, many adsorbents with high specific surface areas and unique shapes are essential for the adsorption strategy. A Zn–Mg–Al/layer double hydroxide (LDH) was designed in this study using a simple co-precipitation process. Adsorbent based on Zn–Mg–Al/LDH was used to remove crystal violet (CV) from the wastewater. The impacts of the initial dye concentration, pH, and temperature on CV adsorption performance were systematically examined. The adsorbents were analyzed both before and after adsorption using FTIR, XRD, and SEM. The roughness parameters and surface morphologies of the produced LDH were estimated using 3D SEM images. Under the best conditions (dose of adsorbent = 0.07 g and pH = 9), the maximum adsorption capacity has been achieved. Adsorption kinetics studies revealed that the reaction that led to the adsorption of CV dye onto Zn–Mg–Al/LDH was a pseudo-second-order model. Additionally, intraparticle diffusion suggests that Zn–Mg–Al/LDH has a fast diffusion constant for CV molecules (0.251 mg/(g min^1/2^)). Furthermore, as predicted by the Langmuir model, the maximal Zn–Mg–Al/LDH adsorption capacity of CV was 64.80 mg/g. The CV dimensionless separation factor (R_L_) onto Zn–Mg–Al/LDH was 0.769, indicating that adsorption was favorable. The effect of temperature was performed at 25, 35, and 45 °C in order to establish the thermodynamic parameters ∆H^o^, ∆S^o^, and ∆G^o^. The computed values indicated exothermic and spontaneous adsorption processes. The study presented here might be used to develop new adsorbents with enhanced adsorption capabilities for the purpose of protecting the water environment.

## Introduction

The generation of wastewater containing dye from numerous industries, including paint, plastics, leather, textiles, pulp and paper, printing, and food industries, is a worldwide problem^1^. The output of dye wastewater has typically increased noticeably in recent years as a result of the rapid expansion of this group of companies. The market today offers more than 100,000 commercial dyes, with annual production exceeding 7 × 10^5^ t^2^. Most industrial dyes are toxic to people and other natural organisms because of their non-biodegradability, chemical stability, and carcinogenic and mutagenic potential^3–5^. Additionally, these dyes significantly affect the aesthetics of water and block sunlight, which has an impact on the photochemical processes in the marine ecosystem^6,7^.

Organic dyes are regarded as the most harmful water pollutants, even at low levels, due to their high toxicity, carcinogenicity, and lack of biodegradability^8,9^. Crystal violet (CV) is one of these dyes that have attracted a lot of interest due to its discharge into water and potential for contaminating water, in addition to its numerous applications in the textile, printing, leather, and coating industries^10^. Since it is mutagenic, carcinogenic, and non-biodegradable, one of the most harmful dyes is crystal violet^11^. It can also remain in the environment for an extended period. Thus, the treatment of CV dyes from wastewater has grown to be a significant field of study. In recent years, a variety of water decolorization techniques have been developed and are being used to remove the colours from industrial effluents. As a result, many techniques have been tried to remove organic dye from contaminated water, including coagulation^12^, photodegradation^13^, chemical oxidation^14^, flocculation^15^, electrodialysis^16^, membrane filtration^17^, and adsorption^18,19^. Adsorption has generated the most interest among the aforementioned techniques because of its greater adsorption capacity, enhanced removal efficacy, environmental friendliness, and low cost^20,21^. Activated carbon, coal, silica gel, activated alumina, fly ash, and metal oxides are a few of the commonly used adsorbents^22,23^. These adsorbents have drawbacks such as poor adsorbent capacity, which results in secondary contamination, a lack of reusability, and other issues that prevent the widespread use of nanostructured adsorbents^24^. Additionally, anionic clays formed of hydrotalcite or layered double hydroxides (LDH) have become a favorable substitute for removing practically all types of harmful pollutants from wastewater^25^.

LDH solves the problems of conventional adsorbents by providing high removal efficiency, an easy synthesis method, high removal of pollutants, recyclability, and affordability^26^. They have also recently received increased interest for a variety of applications, including catalysis^27^, polymer modification^28^, biomedical treatment^29^, and water treatment^30^. LDHs are effective anion exchangers owing to the existence of replaceable interlayer anions^31^. The following general formula shows LDHs: [M^II^_1−x_ M^III^_x_ (OH)_2_]^x+^. [A^n−^_x/n_. mH_2_O], where A^n−^ is an interlayer anion and M^II^ and MI^III^ are the divalent and trivalent metal ions found within the brucite-like layers, respectively^31^. Third metal ions can be added to the binary type of LDH, which can also change the electronic chemical structure and increase electric conductivity, to contribute a large number of active sites with a rapid electron transfer process^32^. Metals such as magnesium, aluminum, and zinc are particularly popular for removing dyes from wastewater^33–35^. The zinc element in LDH influences its morphological and electrochemical properties^36^. Several methods are employed to prepare LDH materials with a variety of physicochemical properties, including co-precipitation^37^, ion-exchange methods^38^, hydrothermal methods^39^, urea hydrolysis^40^, ultrasonic irradiation^41^, and rehydration/reconstruction^42^. The co-precipitation process is one of the synthesis processes that are frequently employed in the literature. It is presented as an easy, inexpensive, and quick procedure that can be quickly scaled up for use in industrial settings^43–45^. It does so in a way that is good for the environment and produces a nanomaterial of high purity without requiring treatments at high temperatures, high pressure, or toxic organic solvents^46^.

To the best of our knowledge, no previous work has been done to remove crystal violet dye using Zn–Mg–Al/LDH. Therefore, the design and construction of Zn–Mg–Al/LDH with excellent adsorption properties is still a challenging task. In this study, crystal violet (CV) was removed from contaminated water using a co-precipitation method to create Zn–Mg–Al/LDH. The microstructure and morphology of the produced samples were examined using Fourier transform infrared spectroscopy (FTIR), scan electron microscopy (SEM), X-ray diffraction (XRD), and energy dispersive X-ray (EDX) spectroscopy. The effects of beginning pH, LDH dosage, initial dye concentration, contact time, and temperature on the removal efficiency of CV dye were investigated using batch research. Isotherm and kinetics models were employed to evaluate the adsorption mechanism and kinetics. The recyclability of Zn–Mg–Al/LDH was examined to determine if the adsorption technique could be made more cost-effective.

## Materials and methods

**Figure 1:**
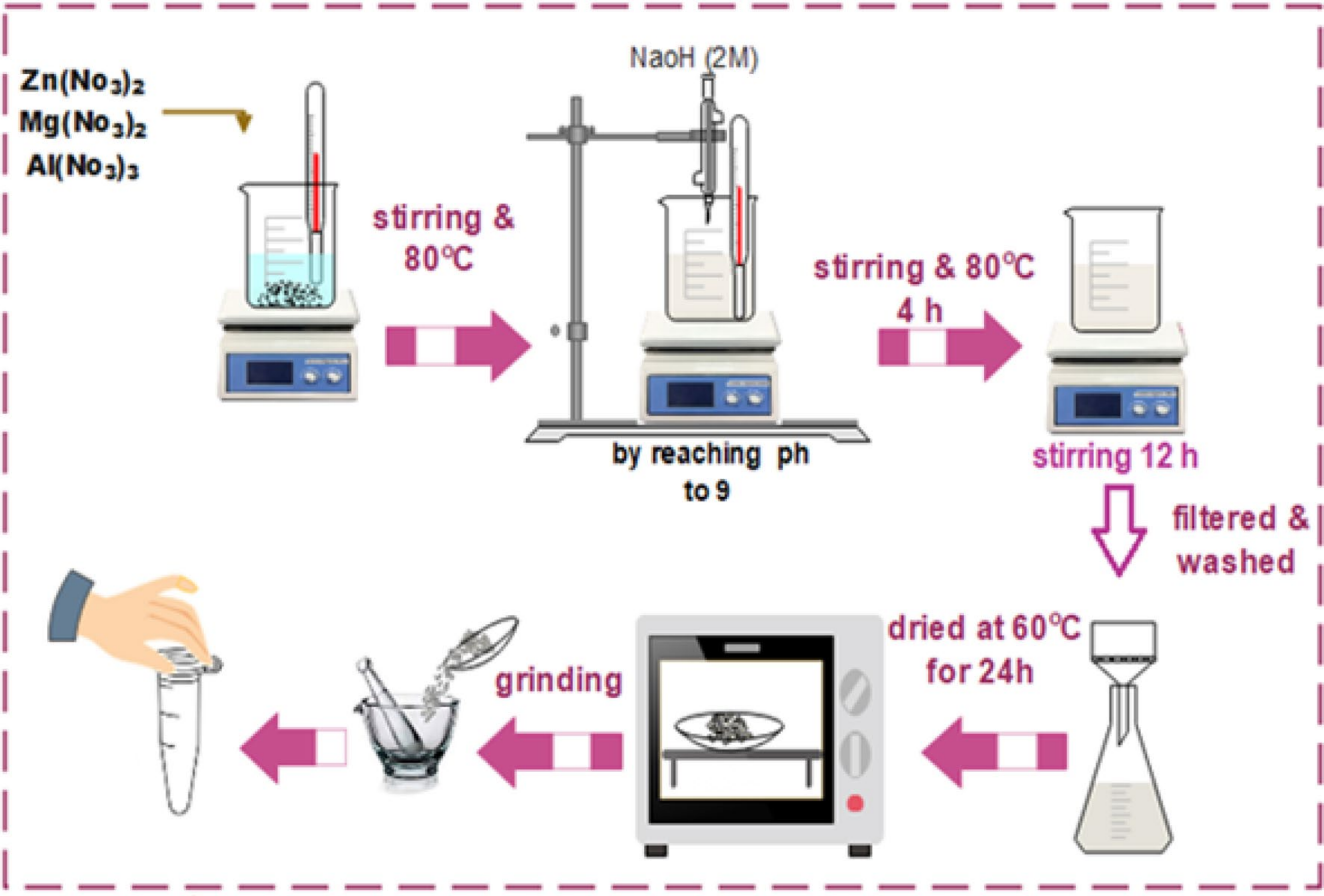
Preparation of Zn–Mg–Al/LDH.

Three different metal nitrates were supplied by LobaChemie, India: magnesium nitrate hexahydrate (Mg (NO_3_)_2_.6H_2_O), zinc nitrate hexahydrate (Zn (NO_3_)_2_.6H_2_O), and aluminum nitrate nonahydrate (Al (NO_3_)_3_.9H_2_O). Crystal violet dye was purchased from Jacquard Products, Canada. Hydrochloric acid and sodium hydroxide were provided by Chemlab NV. The co-precipitation technique was used to prepare the Zn–Mg–Al/LDH. Magnesium nitrate, zinc nitrate, and aluminum nitrate are mixed and dissolved in 100 ml of de-ionized water at a temperature of 80 °C for 4 h with continuous stirring, with a molar ratio of (Zn + Mg): Al = (3:1) and a concentration of Zn:Mg of 0.1 molar ratio. To prevent a fast rise in pH that could lead to the creation of carbonate containing LDH, the precipitate was well mixed, and pH 9 was obtained, by gradually adding NaOH solution (2 mol/L) to the mixture. The mixture was quickly stirred for another 24 h at room temperature (25 °C). The system was then filtered and washed several times with distilled water until the pH equaled 7. The precipitate that formed was then dried for 24 h at 60 °C in a vacuum oven^47^. The Zn–Mg–Al/LDH was then processed to achieve a uniform particle size, as can be demonstrated in Fig. 1.

An X-ray diffractometer (Empyrean X-ray Diffractometer from PANalytical) was used to detect the XRD patterns of LDH before and after adsorption with Cu Kα radiation (λ = 1.5405 Å, 40 kV, 35 mA), using 2θ = 5–80^◦^). An FTIR spectrometer (Bruker Vertex 70) was used to detect the FTIR spectra of LDH and LDH/CV nanopowders. A wide range of wavenumbers between 400 and 4000 cm^−1^ was used to record the FTIR spectra. The morphology of the synthesized LDH and LDH/CV powders was investigated using the SEM (JSM-IT200) after using a sputter coater (JEOL-ION SPUTTER JFC-1100) to deposit gold onto the dispersed powder on a glass slide. The measurements were done at the Central Laboratory for Microanalysis and Nanotechnology, Minia University, Egypt. Using a UV–visible spectrophotometer (Unico instrument-UV2000-USA) to identify and measure the concentration of CV dye still present in polluted water. The UV–Visible absorbance spectra of CV dye at different concentrations in a liquid solution were analyzed using monochromatic electromagnetic radiation in the UV and visible ranges at a wavelength of 585 nm.

The solution’s pH has a considerable effect on the adsorption procedure. The impact of solution pH on dye adsorption was studied while maintaining a constant temperature and a constant concentration of both the dye and the Zn–Mg–Al/LDH adsorbent. Therefore, 0.01 g of Zn–Mg–Al/LDH was put in 25 mL of CV dilution solution (20 ppm) at a specified pH. The values of pH ranging from 3 to 9 were achieved by using 0.1 mol/L NaOH and 0.1 mol/L HCl solutions. Using a composite glass electrode pH meter, the liquids' pH was measured. Filtering was then used to separate the liquid and solid phases. The quantity of CV dye in the liquid solutions was quantified using a visible and ultraviolet spectrophotometer.

The type of surface-active center and the surface's adsorption capacity are both revealed by the pH point of zero charge (pH_pzc_)^48^. The adsorbent's surface has a zero point of charge, PZC, whenever the number of positive charges and the number of negative charges are equal. In order to compute the PZC, 50 mL of deionized water and 5 mg of Zn–Mg–Al/LDH were combined, and the pH was varied from 3 to 9. The solutions were then mixed at 300 rpm for 24 h. The flasks' ultimate pH values were determined. The ∆pH values were plotted against the pH_i_. The PZC value was obtained; this was calculated as the pH equal to zero^49^.

To study the impact of adsorbent dosage, each flask contained 25 mL of CV diluted solution (20 ppm) at pH 9, and the solutions were shaken using a shaker at 300 rpm while the solutions were at room temperature. Different quantities of Zn–Mg–Al/LDH (0.01–0.3 g) were added to each flask. Following that, filtering was used to separate the solid and liquid phases. To investigate the impact of contact time, 25 mL of CV diluted solution (20 ppm) at pH 9 was added to all flasks along with 0.07 g of Zn–Mg–Al/LDH. From 0 to 180 min, at room temperature, a shaker was used to shake the solutions at 300 rpm until equilibrium was established. After a set period, the CV dye solution was filtered to remove the catalyst. The CV dye concentration was then determined using the UV spectrophotometer.

To evaluate the effects of the initial CV concentration, all flasks with initial dye concentrations ranging from 5 to 500 mg/L at pH 9 received 0.07 g of Zn–Mg–Al/LDH. The adsorbents were filtered out of the mixture after the flasks were shaken at 300 rpm at room temperature. The concentrations of residual CV were calculated using the UV spectrophotometer. Under optimal conditions, the impact of temperature on the adsorption procedure was examined. At varying temperatures of 25, 35, and 45 °C, 0.07 g of Zn–Mg–Al/LDH was added to each bottle (25 mL) at pH 9. The bottles were shaken for 120 min at various temperatures. Following the filtration of the Zn–Mg–Al/LDH from the CV solution, using a UV spectrophotometer, the CV concentration was determined.

The reusability of Zn–Mg–Al/LDH is critical for practical applications. If the adsorbent material is recycled, the adsorption process may be more cost-effective and repurposed. In order to process Zn–Mg–Al/LDH, ethanol was used in a number of cycles for both adsorption and desorption, followed by a lot of bi-distilled water for washing. The ethanol washing method was selected owing to its affordability and availability^50^. The cleaned Zn–Mg–Al/LDH after adsorption was dried for 24 h at 60 °C until completely dry. At pH 9, Zn–Mg–Al/LDH is submerged in 20 mg/L of CV solution. Following the adsorption test, the waste was collected, thoroughly cleaned, and then blended for an additional 120 min in a new CV solution (pH 9, 20 mg/L). Five cycles of this procedure were carried out to examine the recyclability of the LDH.

In order to determine the chemical stability of Zn–Mg–Al/LDH, 5 mg of adsorbent was dissolved in 50 mL of HCl or NaOH at pH 9 (because this is the pH at which all experiments have been run), and the mixture was then shaken for 24 h at room temperature. Before measuring the absorbance, the suspension was centrifuged at 3000 rpm for 5 min. The dissolved Zn^+2^ ions in the solution were then measured using an atomic absorption spectrophotometer (AAS) (model ZEISS-AA55, Germany). A hollow cathode lamp (operated at 20 mA) is used as the radiation source with a 2 nm spectral bandpass^51^.

## Results and discussion

### XRD analyses

**Figure 2:**
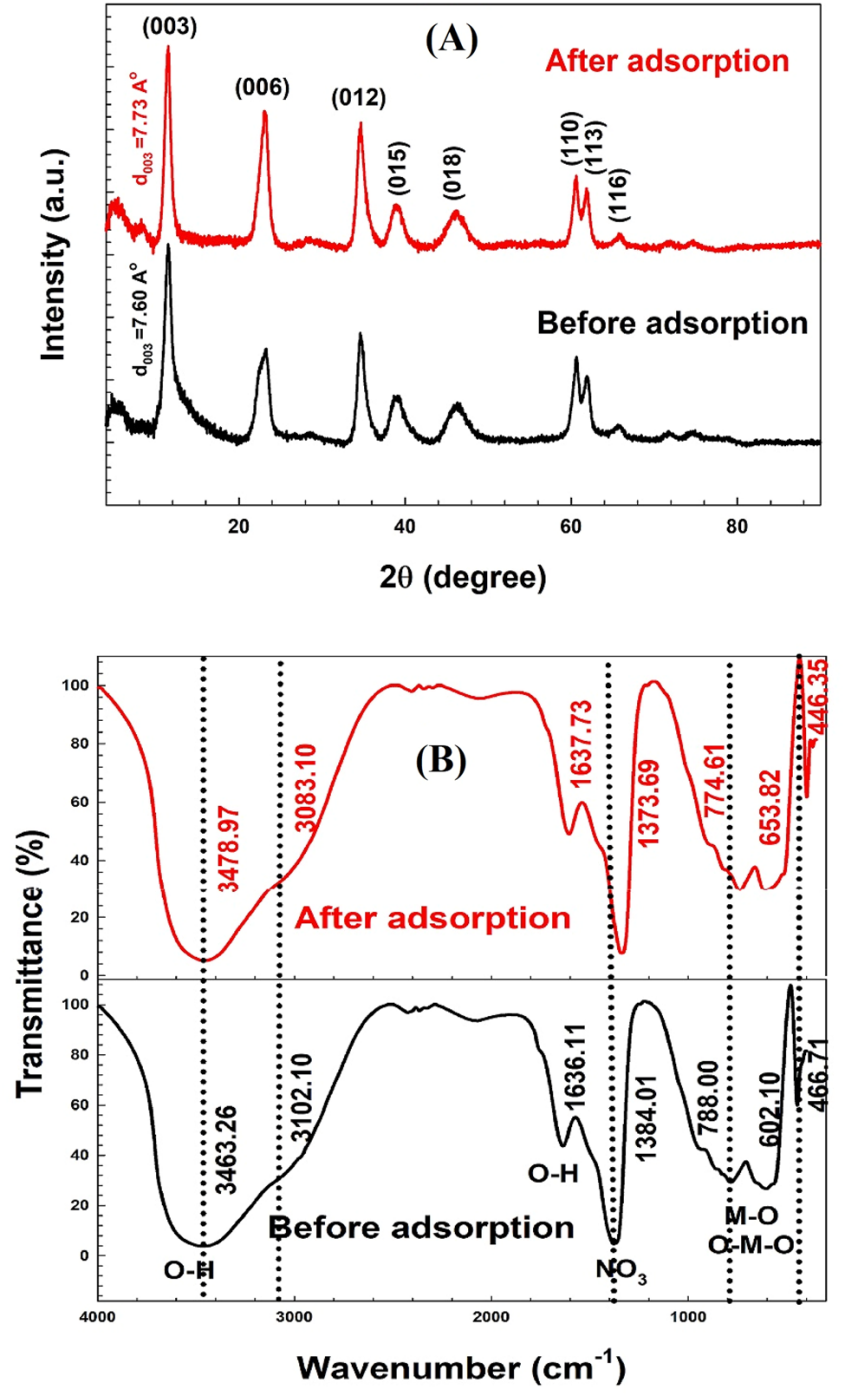
(**A**) XRD patterns and (**B**) FTIR spectra of Zn–Mg–Al/LDH before and after the adsorption of CV.

The X-ray diffraction patterns of the samples are shown in Fig. 2A. The peaks at 11.63◦, 23.38◦, 34.60◦, 38.96◦, 46.31◦, 60.63◦, 61.89◦ and 65.83◦ corresponded to the LDH peaks of (003), (006), (012), (015), (018), (110), (113), and (116), respectively^52–54^. The existence of clear and symmetrical diffraction peaks indicates the high crystallinity of Zn–Mg–Al/LDH, consistent with the ICDD cards (ICDD card No. 48-1023 and ICDD card No. 51-1525). Moreover, for the highest peak intensity at 2θ = 11.63°, the basal spacing is 7.60 Å. The crystalline size of Zn–Mg–Al/LDH was calculated using Deby-Sherrer's formula and found to be 3.60 nm, which is within the accepted range for an LDH structure.After crystal violet (CV) adsorption, the XRD pattern of Zn–Mg–Al/LDH with the basal planes of (003), (006), (012), (015), (018), (110), (113), and (116) at 2θ of 11.43^◦^, 23.19^◦^, 34.72^◦^, 38.96^◦^, 46.14^◦^, 60.63^◦^, 61.89^◦^ and 65.66^◦^, respectively. However, for the highest peak intensity at 2θ = 11.43°, the basal spacing becomes 7.73 Å and the crystalline size is 7.99 nm. Almost all planes are kept in their original positions prior to CV adsorption. This demonstrates that the CV adsorption on Zn–Mg–Al/LDH is a surface adsorption process through surface complication due to the physical and chemical interaction between the LDH surfaces' protonated hydroxyl groups and the CV dye^53,54^.The XRD pattern of Zn–Mg–Al/LDH after crystal violet (CV) adsorption with the basal planes of (003), (006), (012), (015), (018), (110), (113), and (116) at 2θ of 11.43^◦^, 23.19^◦^, 34.72^◦^, 38.96^◦^, 46.14^◦^, 60.63^◦^, 61.89^◦^ and 65.66^◦^, respectively. Though the basal spacing becomes 7.73 Å for the highest peak intensity at 2θ = 11.43° and the crystalline size is 7.99 nm. Almost some planes are kept in their original positions before the adsorption of the CV. This demonstrates that the CV adsorption on Zn–Mg–Al/LDH is a surface adsorption process through surface complication due to the physical and chemical interaction between the LDH surfaces' protonated hydroxyl groups and the CV dye^55,56^.

### FTIR spectra

FTIR spectroscopy was used to further investigate the sample's structure. The prepared Zn–Mg–Al/LDH FTIR spectra before and after crystal violet adsorption are presented in Fig. 2B. The O–H stretching vibration of the water molecule is associated with the broad absorption at 3464.26 cm^−1^^57^. The bending mode of O–H in water molecules is connected to the peak at 1636.11 cm^−1^^58^. The weak shoulder peak, which occurs around 3102.1 cm^−1^, is associated to the OH stretching mode of hydrogen-bonded interlayer anions and interlayer water molecules. Additionally, the presence of NO_3_ anions in the interlayer region is revealed by a strong band at 1377.99 cm^−1^^55^. M–O, O–M–O, and M–O–M lattice vibrations (M = Zn, Mg, and Al) are connected to the bands in the 500–1000 cm^−1^ range^59^. The absorption peak at 445.71 cm^−1^ acts to stretch the vibration of M–OH^60–63^. Figure 2B also includes the FTIR spectrum of the Zn–Mg–Al/LDH after adsorption, which shows that the peak dramatically shifts towards the higher or lower wavenumbers, which prove that crystal violet is adsorbed onto the Zn–Mg–Al/LDH. After CV adsorption, FTIR peak intensities have decreased, which may be due to the intermolecular interactions between the CV and LDH's solid phase.

### SEM analysis

**Figure 3:**
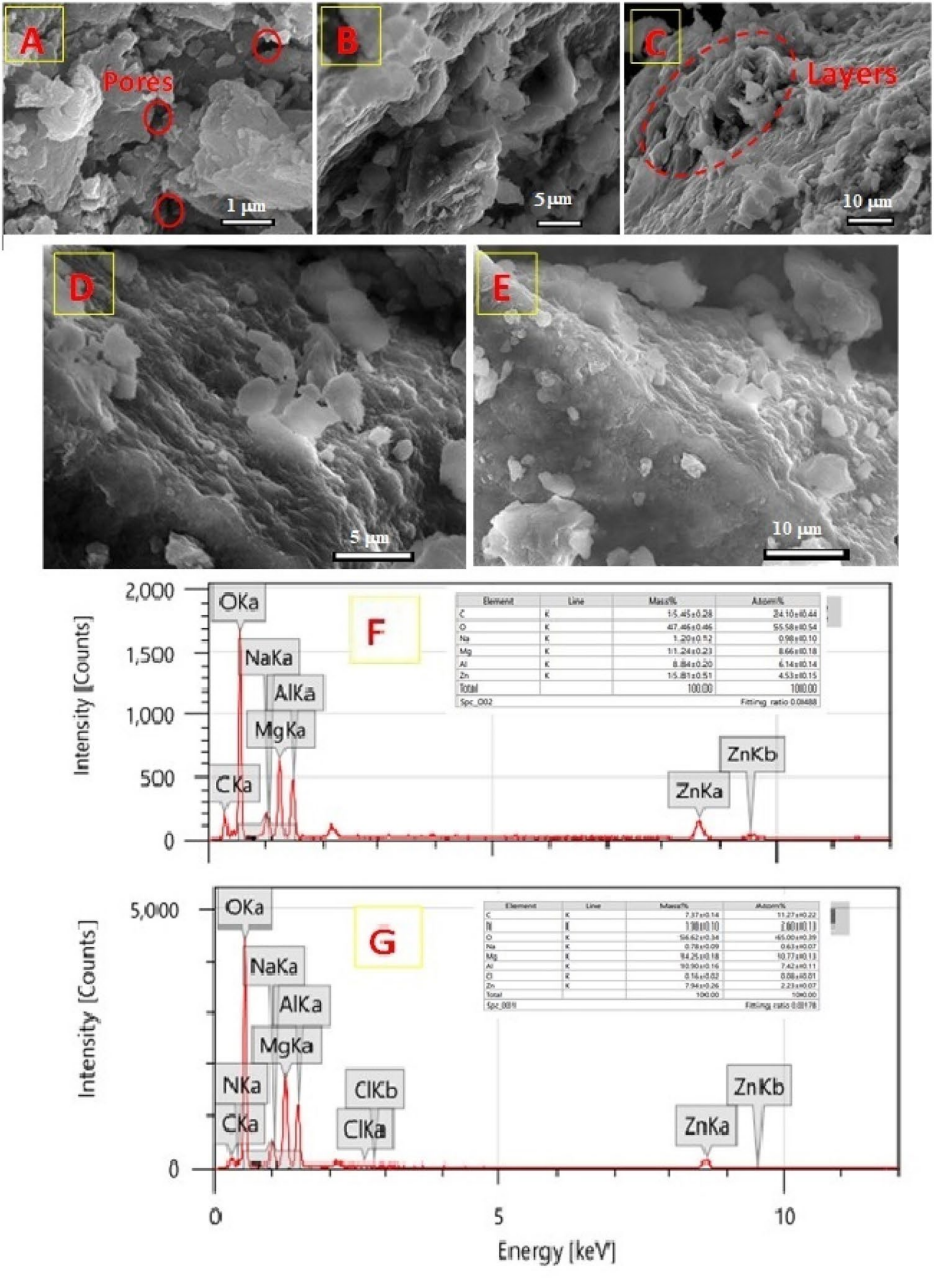
SEM images of Zn–Mg–Al/LDH (**A**–**C**) as prepared, (**D**, **E**) after CV adsorption, (**F**) elemental components of prepared LDH, and (**G**) elemental components of LDH after CV adsorption.

Figure 3A–C display the obtained SEM images of the produced Zn–Mg–Al/LDH, which depicts a plate-like structure and a regularly rough surface that is stacked one on top of the other and has numerous pores. Meanwhile, the surface morphology of the adsorbent changes after the adsorption of CV dye molecule. Figure 3D,E illustrate the surface coverage owing to the adsorption of CV, indicating the good adsorption efficiency of Zn–Mg–Al/LDH. The elemental composition of Zn–Mg–Al/LDH is depicted by the EDX analyses both before and after adsorption, which are presented in Fig. 3F,G, respectively. Weight changes in element atoms after adsorption were also observed, which is clearly because of the dye molecule's adsorption (C_25_H_30_ClN_3_).

### General surface analysis

**Figure 4:**
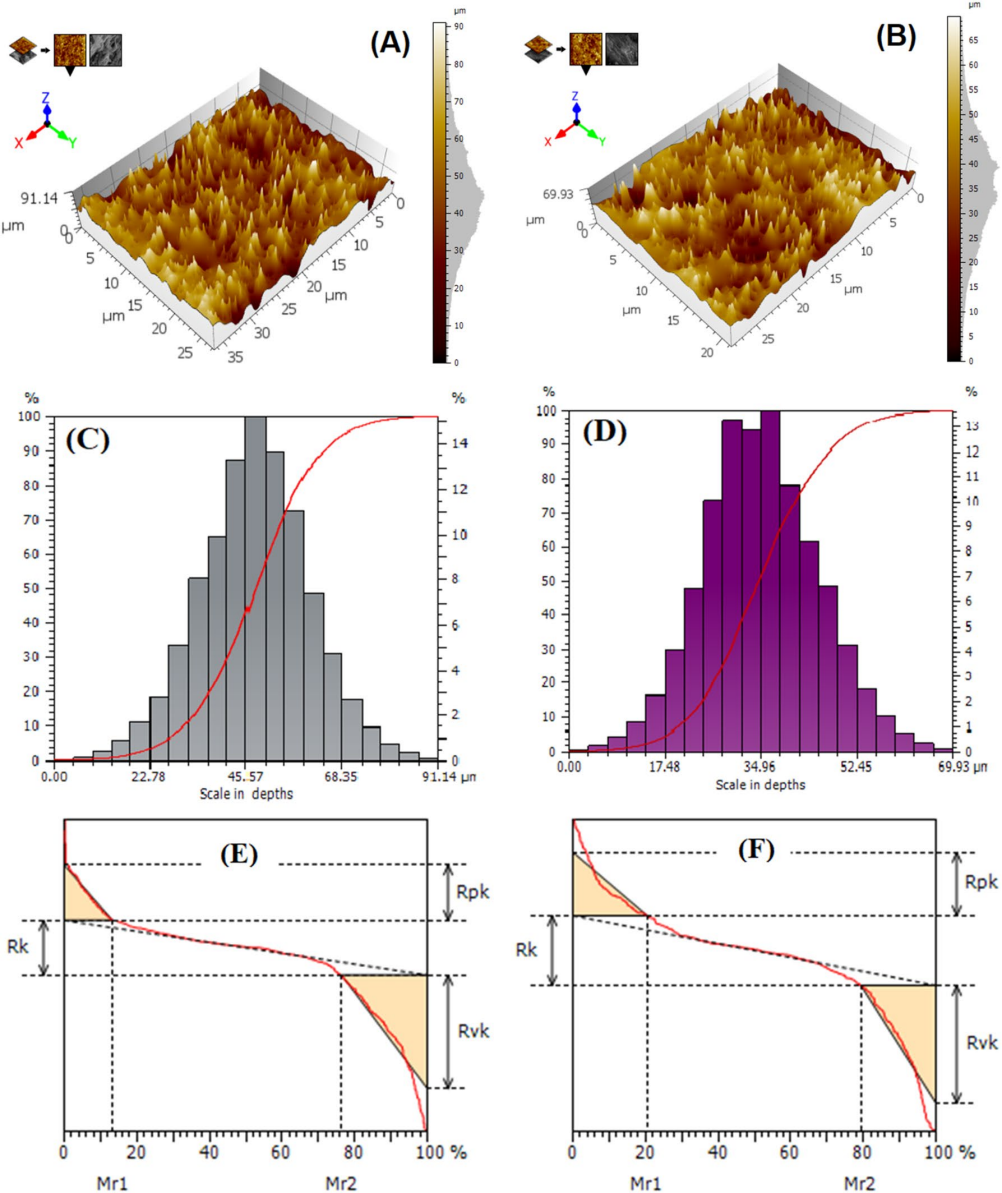
3D images (**A**) before adsorption, and (**B**) after adsorption, the depth histograms and Abbott–Firestone curves (**C**) before adsorption, and (**D**) after adsorption, and schematic representation of a bearing area curve as well as the related roughness parameters (**E**) before adsorption, and (**F**) after adsorption for Zn–Mg–Al/LDH surface.

LDH surface morphology has received a lot of attention due to its ability to show significant characteristics, such as irregularities and heterogeneities, which may have an impact on how the material is used. Contrary to a smooth surface, topographical changes to a surface, such as random roughness, gratings, or isolated bumps, will change the adsorption process. An increase in surface area occurs when the roughness is greatly increased, which is likely to indicate more material has been adsorbed. In principle; roughness can also change the chemical homogeneity of the surface^64^. The topographic SEM image was examined using the Mountain Map® 8.0 software^65^. Figure 4A,B show the representative 3D images of the Zn–Mg–Al/LDH surfaces both before and after adsorption. The analyzed samples' depth histograms and Abbott-Firestone curves are displayed in Fig. 4C,D. The Abbott-Firestone curves show the depth statistical distribution of the sites on the surfaces of the sample, while the histograms provide the distribution density of the sites on the surface^66^. The horizontal axis is computed as a percentage of the total population, whereas the vertical axis refers to the depth. According to this figure, each sample has a distinctive height distribution, and the depth of Zn–Mg–Al/LDH decreases after crystal violet adsorption from 91.14 to 69.93 µm.

A schematic illustration of an S-shaped bearing area curve can be found in Fig. 4E,F. The profile's heights are shown on the vertical axis, and the bearing area lengths are shown on the horizontal axis as a percentage of the profile's overall assessment length^67^. Figure 4E,F also show the roughness characteristics that can be inferred from the bearing area curve, including Mr1, Mr2, Rk, Rpk, and Rvk. The core depth is referred to as the parameter Rk, and it specifies the height of the core material. The value of Rk decreases from 10.52 µm (before adsorption) to 9.85 µm (after adsorption). Reduced peak height, or Rpk, is a parameter that refers to the proportion of peaks above the core profile. The value of Rpk after adsorption decreases from 10.65 to 8.98 µm. The reduced valley depth, or Rvk parameter, refers to the portion of deep valleys that extend into the material beneath the core profile. The value of Rvk decreases from 21.82 µm (before adsorption) to 16.68 µm (after adsorption). The adsorption of dye molecules by LDH causes surface covering, which reduces all roughness parameters. The material ratio at the boundary between protruding peaks and the core is indicated by the parameter Mr1. The material ratio at the transition of the deep valleys and the core is indicated by the parameter Mr2.

**Figure 5:**
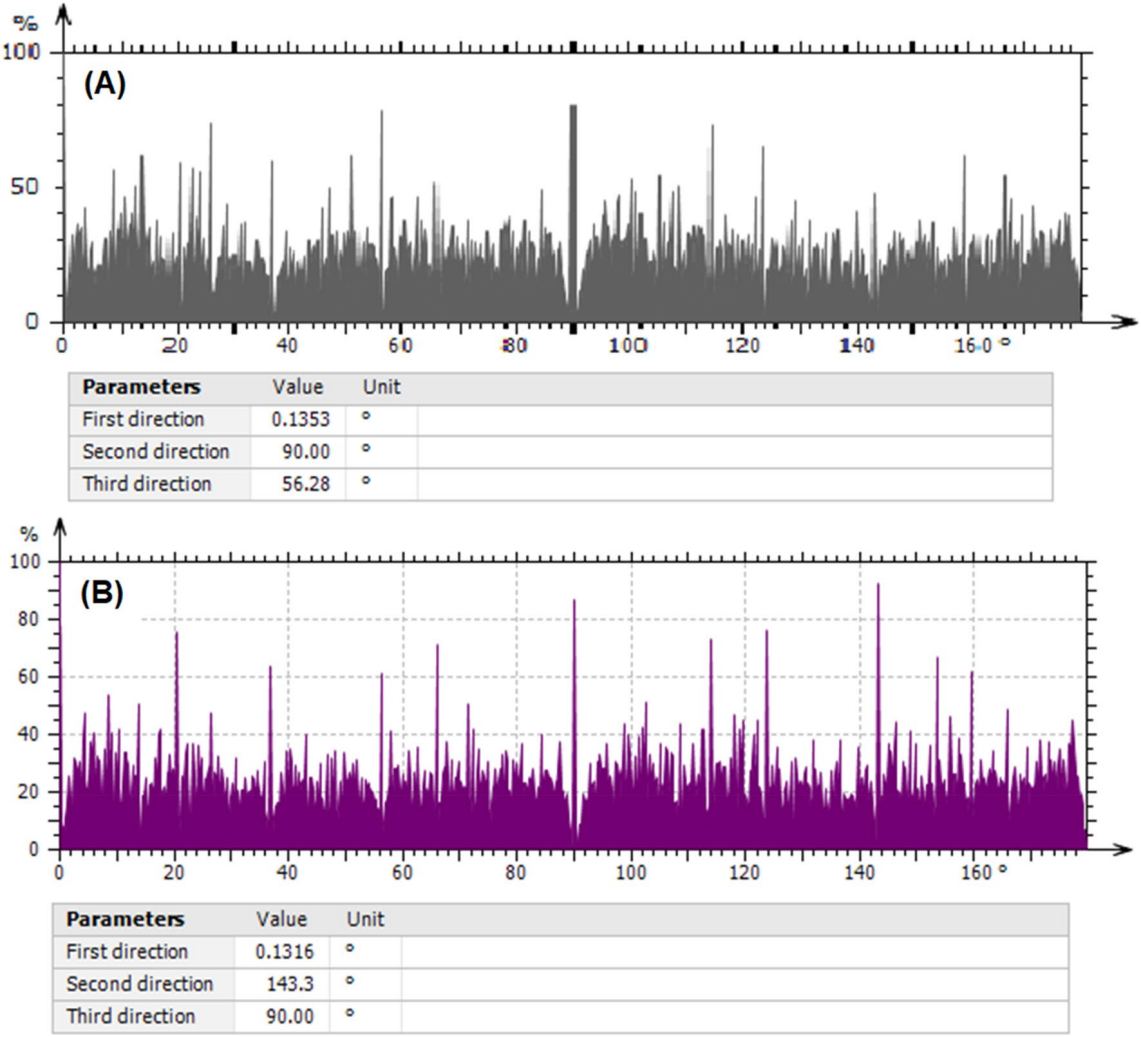
The representation of surface texture directions of analyzed samples using Cartesian graphs for: (**A**) before adsorption, and (**B**) after adsorption.

Cartesian graphs were used to evaluate the samples' surface texture directions. Three preferred angles exist, as depicted in Fig. 5, which might be connected to the surface's inhomogeneity^66^. When the aspect ratio of the texture Str is almost 0, the surface is anisotropic; when Str is nearly 1, the surface is isotropic^68^. In this study, Str before and after adsorption was 0.718 and 0.044, respectively, indicating that the Zn–Mg–Al/LDH surface texture is isotropic. The value of Str is decreased due to loading material on the LDH surface. The parameters of the surface profile, including average roughness Ra, maximum peak to valley height Rt, skewness of the line Rsk, and kurtosis of the line Rku have been calculated. Table 1 lists the surface profile parameters. The amplitude parameters of a sample are explained by factors that give information about statistical average values, the shape of the histogram heights, and other characteristics. The average roughness, Ra, which is widely used to define surface roughness, is determined by taking the mean height throughout the measured area. It's useful for noticing general variations in the profile's overall height properties. Zn–Mg–Al/LDH has a Ra value of 4.03 µm, while Zn–Mg–Al/LDH/CV has a Ra value of 1.35 µm following adsorption, as shown in Table 1. The maximum peak to valley height roughness Rt, which represents the vertical distance between the evaluated area's highest and lowest points and demonstrates the overall roughness of the surface. Zn–Mg–Al/LDH has the Rt value of 25.92 µm, whereas Zn–Mg–Al/LDH/CV has the Rt value of 15.13 µm following adsorption. The dye molecule's adsorption and the LDH surface's coverage clearly account for the decrease in roughness values following adsorption.

**Table 1:** The roughness parameters of prepared LDH before and after adsorption.

Parameters	Symbol	Zn–Mg–Al/LDH	Zn–Mg–Al/LDH/CV
Average roughness	Ra	4.03 µm	1.35 µm
Maximum peak to valley height	Rt	25.92 µm	15.13 µm
Roughness skewness	Rsk	0.237	− 0.242
Roughness kurtosis	Rku	2.740	4.422
Fractal dimensions	Df	1.350	1.344

The roughness skewness Rsk, used to evaluate the symmetry of a surface's variations around the mean plane. The Rsk discusses the features of nonconventional processes, such as porosity and load-carrying ability. Among the Rsk value of 0.237, Zn–Mg–Al/LDH has a peaky surface. Meanwhile, after adsorption, the Zn–Mg–Al/LDH exhibits an Rsk of − 0.242, indicating that the surface of the compound is a valley surface^69^. The roughness kurtosis Rku is applied to calculate the distribution of the peaks above and below the mean plane. For spiky surfaces, Rku > 3; for bumpy surfaces, Rku < 3; perfectly random surfaces have kurtosis = 3^68^. Zn–Mg–Al/LDH has the Rku of 2.740, demonstrating that the surface of Zn–Mg–Al/LDH is a bumpy surface. Meanwhile, Zn–Mg–Al/LDH after adsorption has the Rku of 4.422, demonstrating that the Zn–Mg–Al/LDH/CV surface is spiky. Table 1 also includes a summary of the fractal dimensions Df and correlation coefficient (*R*^*2*^) calculated for the sample surfaces using the enclosing box method. The *R*^*2*^ of the linear fit was equal to 0.996 and 0.998 for Zn–Mg–Al/LDH and Zn–Mg–Al/LDH/CV, respectively; this demonstrates that the data were fit by linear functions very well. The Df is related to the system's complexity^70^. The Df of Zn–Mg–Al/LDH and Zn–Mg–Al/LDH/CV are 1.350 and 1.344, respectively.

### Adsorbent chemical stability

AAS is used to calculate the zinc concentration in solution at pH 9. Zinc plays an important role in the physiological and metabolic processes of many different organisms and is one of the crucial trace elements; however, higher zinc amounts can be hazardous to the organism^71^. Zinc is one of the heavier metals that pose a threat, so it is important to check its chemical stability and make sure it does not decompose. The concentration of zinc in the water samples was 0.058 mg/L and was observed to be below the permissible limits (5 mg/L) as given by the World Health Organization (WHO) and the National Drinking Water Quality Standard (NDWQS)^72,73^. Zn–Mg–Al/LDH exhibits good chemical stability in simulated water.

### Influence of varied factors on adsorption of CV dye

The effectiveness of the adsorption process was assessed using the dye removal efficiency RE (%) and adsorption capacity q_t_ (mg/g) (mg of adsorbate/g of adsorbent) as

1 $$RE\left(\%\right)=\frac{{C}_{o}-{C}_{t}}{{C}_{o}}*100$$

2 $${q}_{t}=\frac{{C}_{o}-{C}_{t}}{m} V$$

Here, *C*_o_ and *C*_t_ are concentrations of dye at time 0 and t, respectively (mg/L), m is the adsorbent's weight (g), and *V* is the solution's volume (L)^74,75^.

#### Influence of initial pH

**Figure 6:**
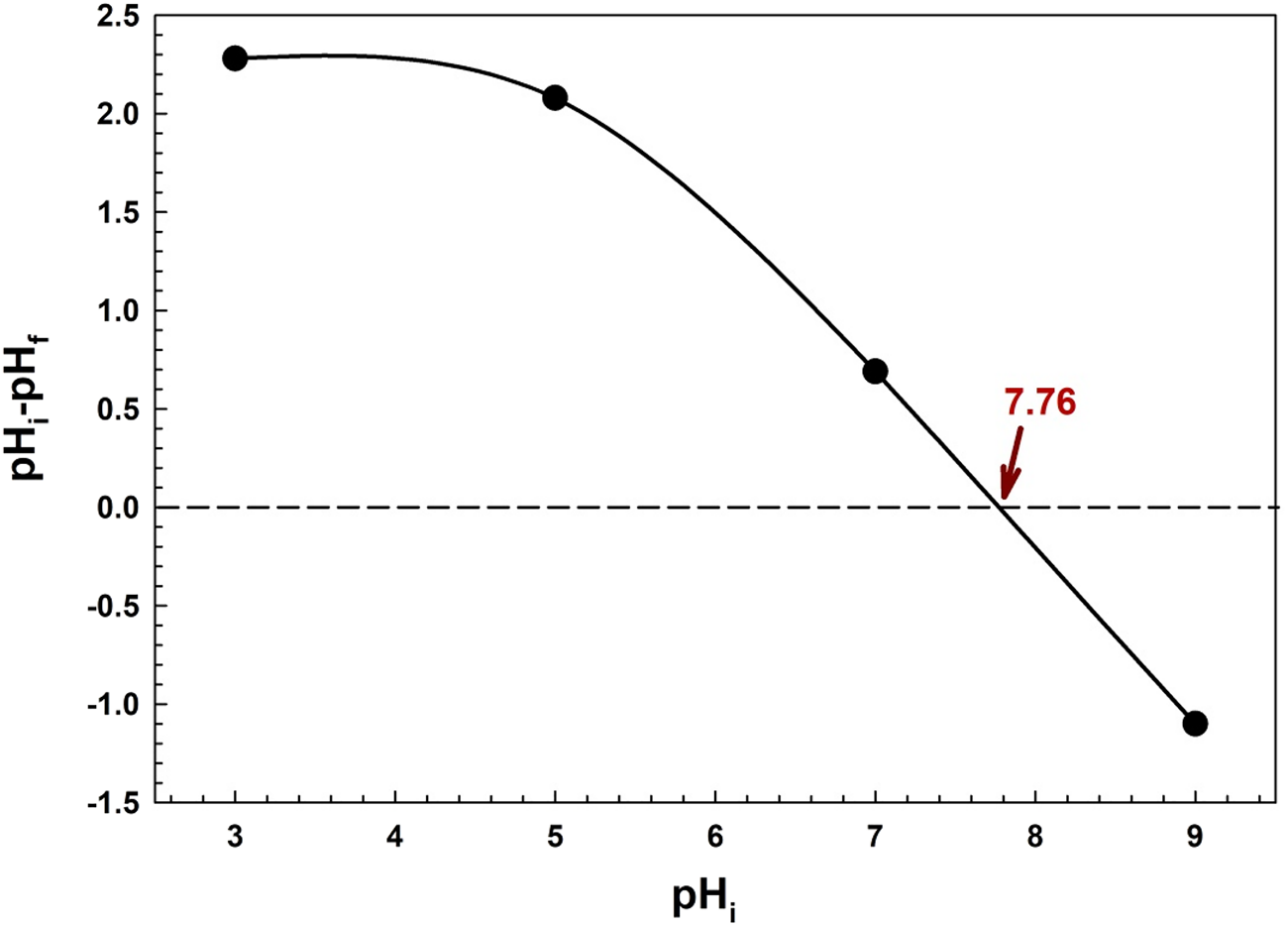
pH point of zero charge of Zn–Mg–Al/LDH.

The pH of the solution has an impact on both the surface charge of the adsorbent and the degree of adsorption of the dye molecule; this significantly affects both the dye's removal from the aqueous solution and its adsorption capacity. At an acidic pH, H^+^ ions and positively charged dye molecules competed for the adsorption sites on the catalyst's surface. At a higher pH, the electrostatic interaction between the negatively charged surface and the cationic dye accelerates the adsorption process, which results in the surface groups becoming deprotonated^76,77^. The point of zero charge (pH_ZPC_) is the pH level of the solution necessary to produce a net zero charge on the surface of the adsorbent. The Zn–Mg–Al/LDH point of zero charge is depicted in Fig. 6. From this plot, ΔpH was 0 at an initial pH value equal to 7.76 (pH_ZPC_ = 7.76). Therefore, when the solution's pH_i_ is greater than 7.76, the Zn–Mg–Al/LDH surface will be negatively charged and be able to attract the cationic dye (CV) via electrostatic interaction.

**Figure 7:**
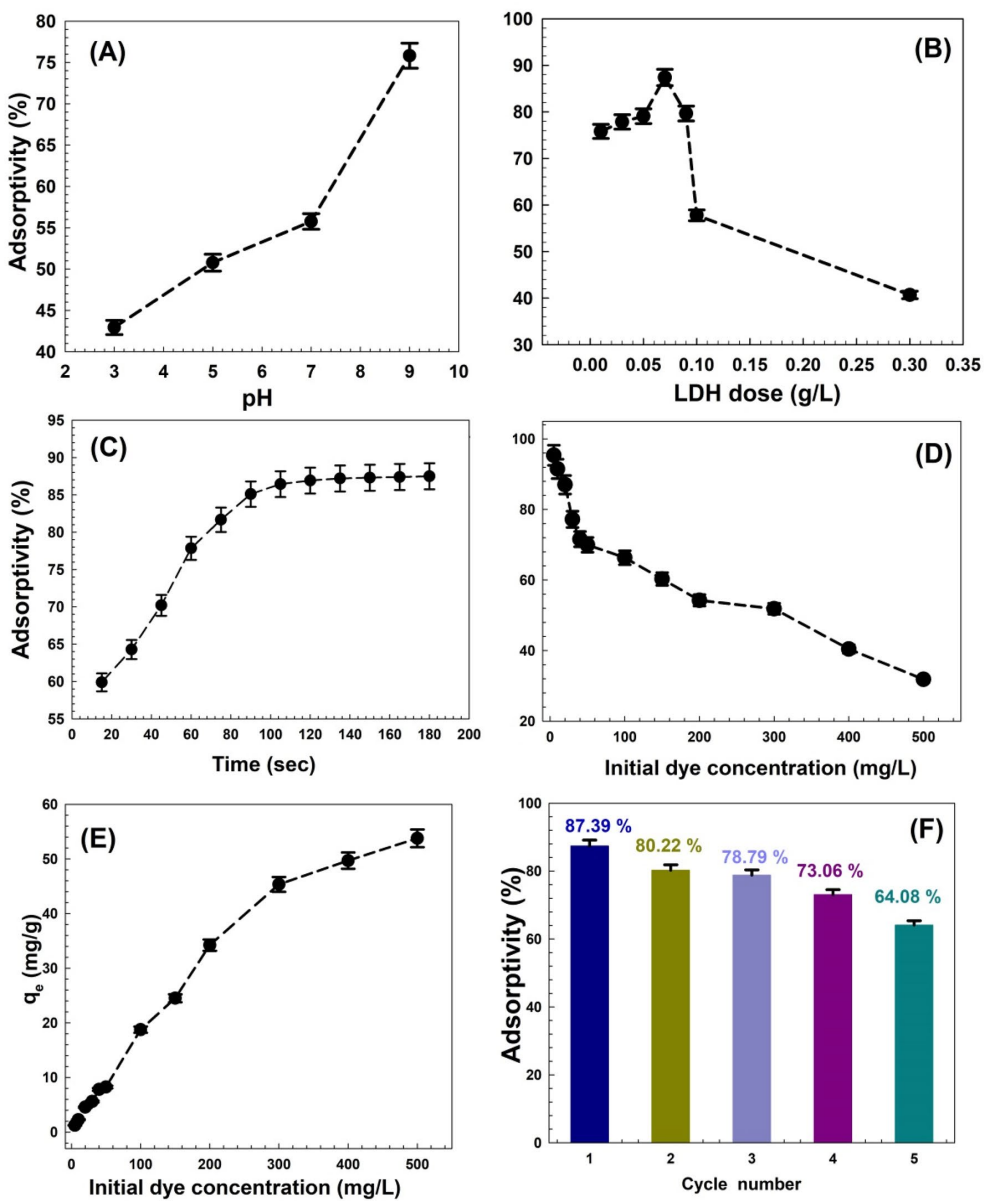
The effect of (**A**) pH solution, (**B**) LDH dose, (**C**) contact time, (**D**) dye concentration, (**E**) initial concentration of CV dye on adsorption capacity, and (**F**) the recyclability of Zn–Mg–Al/LDH on CV adsorption. The smooth short dash curves in the figures are drawn through the data points to guide the eye.

Figure 7A demonstrates the decrease in removal efficiency of CV dye at low pH; at pH 3, the adsorption of CV decreased to 42.93%, which is probably affected by electrostatic repulsion. The removal efficiency sharply increased to 75.81% at pH 9. These data show that variations in pH values have a significant effect on the adsorption of CV as a result of the surface charge changing from positive to negative regions, which enhances the adsorptive process.

#### Influence of adsorbent amount

The adsorbent dose was also simple to regulate during the wastewater treatment process to determine the effectiveness of the adsorbent. Per 25 mL of CV diluted solution at pH 9, an adsorbent dose ranging from 0.01 to 0.3 g was used to measure its effect. Figure 7B depicts the CV removal efficiency results. LDH removal efficiency rose from 75.81 to 86.39% with an additional increase in adsorbent dosage up to 0.01 g. As the adsorbent amount increases, the number of active sites increases, making them more available during adsorption. Whenever the dosage is raised to 0.07 g, the LDH layers bend to aggregate, decreasing the adsorbent surface area and obscuring the active sites^78^. At the dosage of 0.3 g, the removal efficiency decreased to 40.86%.

#### Influence of contact time

Using Zn–Mg–Al/LDH, the impact of contact time on dye removal from an aqueous solution was examined, as demonstrated in Fig. 7C. At early stages, Zn–Mg–Al/LDH was shown to have a fast-rising adsorptivity. For a period of 0 to 100 min, the initial rate of CV adsorption was high and quick. The greater number of activation sites that the adsorbent initially possessed to adsorb CV dye either on the surface or in the interlayer position causes a higher rate of diffusion of CV dye to a solid surface^79^. After 100 min, there was a slight slowdown in the rate of adsorption of the CV dye due to the decreasing CV concentration gradient and the lack of active sites in the adsorbent. At 120 min, the adsorption capacity finally reached saturation.

#### Influence of initial dye concentration

The adsorption capacity is also influenced by the initial concentration of adsorbate, due to its connection to access sites on the adsorbent surface. As the initial concentration rises, the rate at which the adsorption sites become saturated increases. When the concentration is increased, the high-driving forces that are capable of mass transfer also increase the adsorption capacity. The adsorptivity of adsorption is influenced by the amount of CV dye, and its concentration is an important factor in determining the maximum removal concentration at the optimum dosage, pH, and contact time. The concentration range of CV from 5 to 500 mg/L has been selected for this study, as shown in Fig. 7D. This figure shows that as CV concentrations rose from 5 to 500 mg/L, the removal efficiency gradually dropped from 95.3 to 31.84%. This is due to the availability of adsorption sites at the LDH surface at lower concentrations. As the initial concentration was raised to the constant adsorbent dosage (0.07 g), more dye molecules established themselves and filled all the available active sites^80^. At a high CV concentration, there is a lack of free active sites, so dye molecules remain in the solution, which results in a decrease in dye removal. Because of its relationship with available sites on the LDH surface, the initial dye concentration influences adsorptivity. As the CV concentration rises, stronger driving forces become available for mass transfer, increasing the adsorption capacity as a result^81^. Figure 7E illustrates the influence of the initial concentration of CV dye on adsorption capacity. With the increase in the CV concentrations from 5 to 500 mg/L, the adsorption capacity increases from 1.23 to 53.76 mg/g.

#### Influence of recyclability

The experiment examined the regeneration of the Zn–Mg–Al/LDH using an ethanol solution to establish that the adsorbent is a good material for industrial applications. This solution functioned as the adsorbent for primary regeneration after repeated washing. The ability of Zn–Mg–Al/LDH regeneration was investigated at 25 °C and pH = 9 when the initial concentration of crystal violet was 20 mg/L. Figure 7F demonstrated that the removal efficiency of Zn–Mg–Al/LDH was reduced from 87.39 to 64.08% after five regeneration cycles, demonstrating the effective regeneration capabilities of the prepared LDH. It is obvious that the reduction in adsorption capacity causes the adsorption efficiency to decrease with an increase in cycle numbers. After the fifth cycle of usage, adsorption performance could be impacted by changes in the adsorbent's chemistry and structure, as well as by changes in the mass transport conditions^82^.

### Adsorption isotherm models

The isotherm is a key aspect for actually describing the adsorbate-adsorbent interaction. At a given temperature, the dynamic equilibrium relation between the concentration of the adsorbate and adsorption capacity is demonstrated by the isotherm curve. The Langmuir and Freundlich models, two well-known isotherms, were employed to simulate the solid–liquid adsorption procedure. According to the Langmuir isotherm model, the adsorption process takes place in a monolayer on a homogenous surface^83^. The Langmuir adsorption isotherm model's non-linear form can be expressed as

3 $${q}_{e}={q}_{\mathrm{max}}\frac{{{\mathrm{K}}_{\mathrm{L}}{\mathrm{C}}_{\mathrm{e}}}_{ }}{1+{\mathrm{K}}_{\mathrm{L}}{\mathrm{C}}_{\mathrm{e}}}$$

Here, *q*_e_ (mg/g) and *C*_e_ (mg/L) are the adsorption capacity and the equilibrium concentration of the adsorbate, respectively. *q*_max_ (mg/g) is the maximum adsorption capacity, while K_L_ with(L/mg) is the Langmuir constant (indicates the affinity of the adsorbate for the active sites). The nature of the adsorption procedure can be predicted by the dimensionless separation factor (R_L_) as

4 $${\text{R}}_{{\text{L}}} = \frac{1}{{1 + K_{L} C_{0} }}$$

When R_L_ = 0 suggests that the process of adsorption is irreversible. Adsorption moves forward favorably at 0 < R_L_ < 1, while becoming unfavorable at R_L_ > 1. The adsorption process is demonstrated to have a linear relationship when R_L_ = 1^84^.

The Freundlich isotherm describes a heterogeneous surface with non-ideal and reversible adsorption on active sites with an exponential energy distribution^85^. The Freundlich adsorption isotherm could be represented in its non-linearized form as

5 $${{q}_{e}=K}_{f}.{{C}_{e}}^{1/n}.$$

where *n* is the intensity factor, and *K*_*F*_ (mg/g) (L/mg)^1/n^ is the Freundlich constant for the adsorption capacity. The value of *n* indicates whether the adsorption is difficult (*n* < 1), partially difficult (1 ≤ *n* < 2), or easy (2 ≤ *n* < 10)^86^.

**Figure 8:**
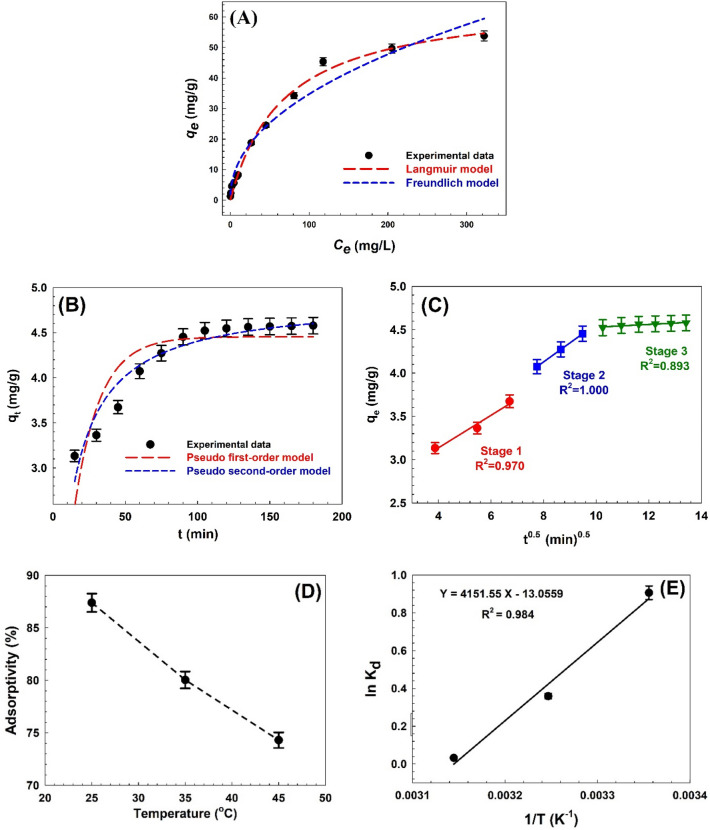
(**A**) Experimental isotherm models, (**B**) Experimental kinetic models, and (**C**) the diffusion model, (**D**) Effect of tempeture, and (**E**) adsorption thermodynamics of CV adsorption on Zn–Mg–Al/LDH.

Using two isothermal non-linear adsorption models, Fig. 8A shows the fit of the experimental data to the equilibrium isothermal Langmuir and Freundlich models. In comparison to linear regression, the non-linear regression approach was found to be superior for obtaining the isotherm parameters and selecting the ideal isotherm^87^. Table 2 summarizes the parameters of the two different models. The Langmuir and Freundlich isotherm models of CV adsorption onto Zn–Mg–Al/LDH adsorbent were obtained to have correlation coefficients (*R*^*2*^) of 0.996 and 0.983, respectively, indicating that the Langmuir and the Freundlich isotherm models provide a more complete explanation for the adsorption of CV dye onto the Zn–Mg–Al/LDH adsorbent.

**Table 2:** The isotherm models and their parameters obtained from the fitting results.

Isotherm models	Parameters	Values of parameters
Langmuir isotherm model	*K*_L_ (L/mg)	0.015
	*Q*_max(cal)_ (mg/g)	64.80
	*R*_L_	0.769
	*R*^2^	0.996
Freundlich isotherm model	*K*_f_ (mg/g) (L/mg)^1/n^	4.176
	1/*n*	0.460
	*R*^2^	0.983

The separation factor (*R*_*L*_) provides a comprehensive explanation for the Langmuir equilibrium data. It was determined that CV was adsorbed onto Zn–Mg–Al/LDH, and the R_L_ value (0.769) was less than 1 and more than 0, demonstrating that that adsorption was favorable. The strong adsorption of the adsorbents towards CV molecules is shown by the low R_L_ value^76^. According to Table 2, the Zn–Mg–Al/LDH maximum adsorption value for CV was 64.80 (mg/g). The system fitted well to the Freundlich isotherm with a (1/*n*) of 0.460 mg/L, which described the favorable adsorption conditions and the degree of LDH surface heterogeneity.

### Adsorption kinetic models

To estimate the adsorption mechanism, kinetic and isotherm models can be analyzed in both linear and non-linear modes^88^. To minimize errors, the experimental results were examined using kinetic and isotherm models based on non-linear ways to investigate the adsorption's controlling processes^89^. By fitting the experimental data to the Weber-Morris intraparticle diffusion model (IPD), the Lagergren pseudo-first-order kinetic model (PFO), and the pseudo-second-order kinetic model (PSO)^90^. The PFO model is useful for demonstrating a mathematical correlation between the adsorption rate and the adsorbed mass as follows:

6 $$q_{t} = q_{e} (1 - e^{{ - K_{1} t}} ).$$

The PFO adsorption rate constant is *K*_*1*_ (min^−1^), and *q*_e_ and *q*_t_ (mg/g) represent the amount of solute adsorbed at equilibrium and at any time t, respectively.

The PSO model predicts that the adsorption process will be controlled by chemisorption, which includes valence forces produced by the sharing or exchanging of electrons between the adsorbed species and the adsorbent^91^. The kinetics of PSO can be expressed as.

7 $$q_{t} = \frac{{{\text{q}}_{{\text{e}}}^{2} {\text{K}}_{{2{ }}} {\text{t}}}}{{1 + {\text{q}}_{{\text{e}}} {\text{ K}}_{2} {\text{t}}}}.$$

Here, *K*_*2*_ (g/(mg min)) is the PSO adsorption rate constant.

The CV adsorption mechanism is depicted in Fig. 8B, employing a variety of kinetic models, including intra-particle diffusion, pseudo-second-order reaction (PSO), and pseudo-first-order reaction (PFO). The *R*^*2*^ values from the PFO (0.750) and PSO (0.920) models are shown to be comparable in Table 3. Due to this, it can be determined that the adsorption kinetics data that were obtained experimentally may adhere more closely to the PSO model than to the PFO model. Furthermore, the calculated adsorption capacity *q*_*calc*_ is 4.87 mg/g, which is very similar to the experimental adsorption capacity *q*_*exp*_ which was 4.57 mg/g, as revealed in Table 3. Thus, the better fit of the PSO model may indicate that CV dye adsorption on the Zn–Mg–Al/LDH composite is controlled by chemisorption rather than physisorption.

**Table 3:** The adsorption kinetic models and their parameters obtained from the fitting results.

Kinetic models	Parameters	Values of parameters
Pseudo-first- order	*K*_1_ (min^−1^)	0.057
	*q*_e (cal)_ (mg/g)	4.455
	*R*^2^	0.750
Pseudo-second-order	*K*_2_ (g/(mg min))	0.019
	*q*_e (cal)_ (mg/g)	**4.872**
	*R*^2^	0.920
Intraparticle diffusion	*K*_ip_ (mg/(g.min^1/2^))	0.251
	*C*_ip_ (mg/g)	2.38
	*R*^2^	1.00
Experimental data	*q*_e (exp)_ (mg/g)	**4.57**

Significant are in value [bold].

The Weber-Morris Intraparticle-diffusion (IPD) model can be used to determine the potential rate-controlling step in the dye adsorption process as

8 $$q_{e} = K_{ip} \sqrt t + C_{ip} .$$

The intra-particle rate constant is described as *K*_*ip*_ (mg/(g min^1/2^)), and the boundary layer thickness is mentioned to *C*_*ip*_ (The boundary layer effect increases with increasing C_ip_ values). The likelihood that the solute will diffuse within the adsorbent pores increases with the *K*_*ip*_ value.

Figure 8C shows how Intraparticle-diffusion works. The dye adsorption onto an adsorbent is said to take place in multiple stages, such as adsorption on the surface of the adsorbent, diffusion within the particles (intercalation), and saturation. CV dye adsorption on Zn–Mg–Al/LDH was controlled by multiple steps in the intraparticle diffusion plot at various time points. The diffusion adsorption stage, represented by the first linear step, is the diffusion adsorption of CV on the Zn–Mg–Al/LDH external surface under the driving force of the solution. Under the effect of the driving force of intramolecular mass transfer, the second step depicts CV's intra-particle diffusion through the pores of Zn–Mg–Al/LDH. The equilibrium adsorption stage, in which LDH achieved adsorption equilibrium as a result of internally optimized coordination, is described in the final linear step^84^. Additionally, the boundary layer thickness *C*_*ip*_ and the intraparticle diffusion constant *K*_*ip*_ were estimated using the plot's second linear segment^92^ and are listed in Table 3.

### Influence of temperature and calculation of thermodynamic parameters

The important thermodynamic parameters, such as the enthalpy change (ΔH◦), entropy change (ΔS◦), and free energy change (ΔG◦), can be adjusted to obtain a more accurate result, to establish the possibility of adsorbate-adsorbent interactions as^93^.

9 $$\ln K_{d} = \frac{{\Delta S^{o} }}{R} - \frac{{\Delta H^{o} }}{RT},$$

10 $$\Delta G^{o} = - RT\ln K_{d} .$$

*T* is the solution's absolute temperature (K), *R* is the universal gas constant (8.314 J/mole K), and *K*_*d*_ = *q*_*e*_/*C*_*e*_ is the thermodynamic equilibrium constant (L/g). Batch adsorption experiments were also carried out with 0.07 g of the Zn–Mg–Al/LDH composite and 25 mL of the 20 mg/L dye solution at a pH of 9 to assess the thermodynamic parameters. The impact of temperature on Zn–Mg–Al/LDH adsorption efficiency during CV adsorption is shown in Fig. 8D. As the temperature increased from 25 to 45 °C, the adsorptivity decreased from 87.39 to 74.30%, demonstrating that the CV dye's interaction with Zn–Mg–Al/LDH is exothermic in nature^94^. Through hydrogen bonding and attraction forces, the CV dye is adsorbed onto the Zn–Mg–Al/LDH. Therefore, the adsorption efficiency of Zn–Mg–Al/LDH decreases as the temperature rises.

The thermodynamic parameters that are impacted by the temperature dependence of the adsorption process are the Gibbs free energy change ΔG^o^, enthalpy ΔH^o^, and entropy ΔS^o^ of adsorption. These parameters were calculated using Eqs. (9) and (10). The slope and offset of the ln *K*_*d*_ versus 1/*T* curve were used to calculate the ΔH◦ and ΔS◦ values, as demonstrated in Fig. 8E. The thermodynamic parameters' outcomes are listed in Table 4. Therefore, the spontaneity and viability of the adsorption process are demonstrated by the negative ΔG◦ values at all of the temperatures tested^95^. When the temperature was raised from 298 to 318 K, the values of ΔG^o^ enhanced from − 2.24 to − 0.085 kJ/mol, proving that at lower temperatures, the dye adsorption process happens more spontaneously^94^. The exothermic nature of CV dye adsorption by the Zn–Mg–Al/LDH is also supported by the negative ΔH◦ value^96^. A negative ΔS◦ value shows that the molecules of dye adsorb in a systematic manner at the solid–liquid interface (a reduction in the amount of randomness at the dye molecules' adsorption)^97^.

**Table 4:** Thermodynamic parameters for the adsorption of CV dye by Zn–Mg–Al/LDH.

Temperature (K)	ΔG^o^ (kJ/mol)	∆H^o^ (kJ/mol)	∆S^o^ (J/(mol K))
298	− 2.240	− 34.515	− 108.54
308	− 0.919		
318	− 0.085		

### Possible adsorption mechanism

**Figure 9:**
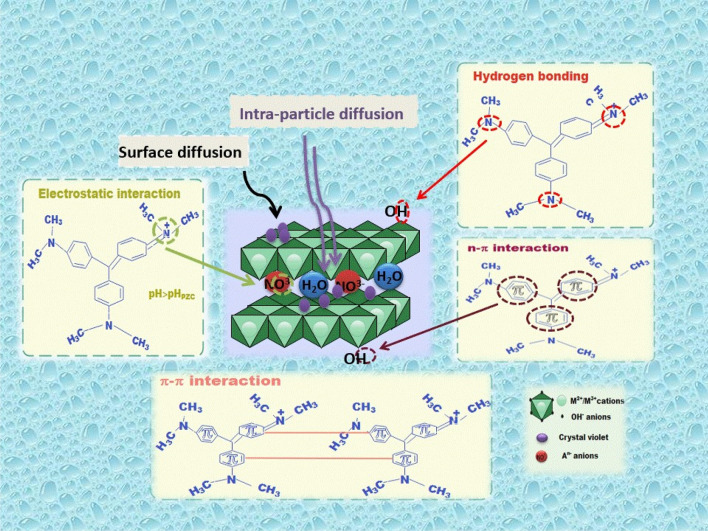
Possible adsorption mechanism of Zn–Mg–Al/LDH.

For a complete understanding of the process, an explanation of the adsorption mechanism is necessary. The nature of the adsorbate, its structural and functional groups, its surface and textural characteristics, dye diffusion, and the type of interaction between the dye molecules and the LDH are all important aspects of the adsorption process^98^. Zn–Mg–Al/LDH's potential adsorption interactions with CV dye are presented in detail in Fig. 9. The CV dye adsorbent's Zn–Mg–Al/LDH adsorption can be influenced by hydrogen bonding, electrostatic attraction, n–π interaction, π–π interaction, mesoporous filling, and surface diffusion. The hydrogen bond that exists between the nitrogen in the CV dye (H-acceptors) and the hydrogen of the hydroxyl groups (H-donors) is noted^99^. The CV-adsorbed sample's FTIR spectrum shows a sharp drop in the OH group intensity and shifts towards the somewhat higher wavenumbers of 3478.97 cm^−1^, indicating proof of the presence of dipole–dipole hydrogen bonding^100^. Through the n- interaction, the OH or oxygen bonds on the surface of Zn–Mg–Al/LDH and the aromatic ring in the CV dye interact^101^. When CV aromatic rings interact non-covalently, the π–π interaction takes place^102^.

Electrostatic attraction mechanisms are usually explained in terms of the interaction between the cationic CV and sites with negative charges on the Zn–Mg–Al/LDH surface when the pH solution > pH_PZC_ (pH solution > 7.76)^103^. The CV-adsorbed sample's FTIR spectrum shows that the peak around 1384.01 cm^−1^ shifts towards the wavenumber of 1373.69 cm^−1^, demonstrating the electrostatic interaction between the positively charged CV and the negatively charged nitrate ions. The surface properties of Zn–Mg–Al/LDH and the adsorption capability, which is directly proportional to the pore surface area, were correlated with the pore-filling mechanism (intra-particle diffusion) and surface diffusion of Zn–Mg–Al/LDH. The material's high surface areas and pore volumes often encouraged the adsorption of organic pollutants because of the strong pore-filling effect^104^.

## Conclusion

Water pollution is currently recognized as a major issue on a global scale, and several initiatives are being taken to mitigate its effects. Additionally, water contains numerous pollutants, such as dyes and heavy chemical elements, among others. To remove CV from a polluted solution, Zn–Mg–Al/LDH was prepared in this study through co-precipitation. The co-precipitation method resulted in the successful production of a Zn–Mg–Al/LDH nanoparticle with a structure that was thoroughly characterized by XRD, FTIR, and SEM. Zn–Mg–Al/LDH has the maximum peak to valley height *R*_*t*_ value of 25.92 µm, whereas Zn–Mg–Al/LDH has *R*_*t*_ value of 15.13 µm following adsorption. After adsorption, the dye molecule and the LDH surface adsorb, which clearly results in a decrease in roughness values. The aqueous solution of CV dye was well absorbed by the synthesized Zn–Mg–Al/LDH nanoparticles, which had an excellent adsorption capacity of 64.80 mg/g. At pH = 9 and an adsorbent dose of 0.07 g, the Zn–Mg–Al/LDH nanoparticles had an adsorptive capacity of 87.3%. The pseudo-second-order kinetic fit for the adsorption kinetics was better than the pseudo-first-order fit, indicating that the chemisorption process dominated the adsorption mechanism. In addition, according to intraparticle diffusion, the CV adsorption process onto Zn–Mg–Al/LDH involves several steps, including diffusion within the particles (intercalation), adsorption onto the adsorbent's active sites (saturation), and adsorption on the surface of the adsorbent. The adsorption isotherm was fit using the Freundlich and Langmuir isotherm models. Both models were better at representing the homogenous multilayer adsorption of CV dye on the Zn–Mg–Al/LDH adsorbent. Thermodynamic parameters indicated that CV adsorption on Zn–Mg–Al/LDH was exothermic and spontaneous. After five cycles, Zn–Mg–Al/LDH's removal efficiency for CV dropped to 64.08%.

## Data Availability

The datasets used and/or analyzed during the current study available from the corresponding author on reasonable request.

## Abbreviations

AAS: Atomic absorption spectrophotometer
C_ip_: Boundary layer thickness
C_o_: Concentration of dye at time 0
C_t_: Concentration of dye at time t
CV: Crystal violet
Df: Fractal dimensions
FTIR: Fourier transform infrared spectroscopy
IPD: Intraparticle diffusion model
K_d_: Equilibrium constant
K_F_: Freundlich constant
K_ip_: Intraparticle diffusion constant
K_L_: Langmuir constant
LDH: Layer double hydroxide
M: Weight of adsorbent
NDWQS: National drinking water quality standard
PFO: Pseudo-first-order kinetic model
pH_ZPC_: PH at Point of zero charge
PSO: Pseudo-second-order kinetic model
q_e_: Amount of solute adsorbed at equilibrium
Qt: Adsorption capacity
R: Ideal gas constant
Ra: Average roughness
RE: Removal efficiency
Rk: Core depth
Rku: Kurtosis of the line
R_L_: Separation factor
Rpk: Reduced peak height
Rsk: Skewness of the line
Rt: Maximum peak to valley height
Rvk: Reduced valley depth
SEM: Scanning electron microscopy
Str: Aspect ratio of the texture
V: Volume of the test solution
WHO: World health organization
XRD: X-ray diffraction
ΔG◦: Free energy change
ΔH◦: Enthalpy change
ΔS◦: Entropy change

## References

[1] Chowdhury S, Pan S, Balasubramanian R, Das P, Naushad M, Lichtfouse E (2019). Date palm based activated carbon for the efficient removal of organic dyes from aqueous environment. Sustainable Agriculture Reviews.

[2] Khalid A, Zubair M (2018). A comparative study on the adsorption of Eriochrome Black T dye from aqueous solution on graphene and acid-modified graphene. Arab. J. Sci. Eng..

[3] El Gaini L, Lakraimi M, Sebbar E, Meghea A, Bakasse M (2009). Removal of indigo carmine dye from water to Mg–Al–CO_3_-calcined layered double hydroxides. J. Hazard. Mater..

[4] Al-Momani F, Touraud E, Degorce-Dumas JR, Roussy J, Thomas O (2002). Biodegradability enhancement of textile dyes and textile wastewater by VUV photolysis. J. Photochem. Photobiol. A.

[5] Zare K, Gupta VK, Moradi O, Makhlouf ASH, Sillanpää M, Nadagouda MN, Kazemi M (2015). A comparative study on the basis of adsorption capacity between CNTs and activated carbon as adsorbents for removal of noxious synthetic dyes: A review. J. Nanostruct. Chem..

[6] Buvaneswari N, Kannan C (2011). Plant toxic and non-toxic nature of organic dyes through adsorption mechanism on cellulose surface. J. Hazard. Mater..

[7] Nasuha N, Hameed BH, Din ATM (2010). Rejected tea as a potential low-cost adsorbent for the removal of methylene blue. J. Hazard. Mater..

[8] Yaseen DA, Scholz M (2018). Treatment of synthetic textile wastewater containing dye mixtures with microcosms. Environ. Sci. Pollut. Res..

[9] Alhogbi BG, Altayeb S, Bahaidarah EA, Zawrah MF (2021). Removal of anionic and cationic dyes from wastewater using activated carbon from palm tree fiber waste. Processes.

[10] Zhang Q, Zhang T, He T, Chen L (2014). Removal of crystal violet by clay/PNIPAm nanocomposite hydrogels with various clay contents. Appl. Clay Sci..

[11] Shoukat S, Bhatti HN, Iqbal M, Noreen S (2017). Mango stone biocomposite preparation and application for crystal violet adsorption: A mechanistic study. Microporous Mesoporous Mater..

[12] Luo X, Liang C, Hu Y (2019). Comparison of different enhanced coagulation methods for azo dye removal from wastewater. Sustainability.

[13] Liu CC, Hsieh YH, Lai PF, Li CH, Kao CL (2006). Photodegradation treatment of azo dye wastewater by UV/TiO_2_ process. Dyes Pigm..

[14] Zou H, Ma W, Wang Y (2015). A novel process of dye wastewater treatment by linking advanced chemical oxidation with biological oxidation. Arch. Environ. Prot..

[15] Moghaddam SS, Moghaddam MA, Arami M (2010). Coagulation/flocculation process for dye removal using sludge from water treatment plant: Optimization through response surface methodology. J. Hazard. Mater..

[16] Lafi R, Gzara L, Lajimi RH, Hafiane A (2018). Treatment of textile wastewater by a hybrid ultrafiltration/electrodialysis process. Chem. Eng. Process.-Process Intensif..

[17] Liu X, Xiao Y, Zhang Z, You Z, Li J, Ma D, Li B (2021). Recent progress in metal–organic frameworks@ cellulose hybrids and their applications. Chin. J. Chem..

[18] Elhalil A, Qourzal S, Mahjoubi FZ, Elmoubarki R, Farnane M, Tounsadi H, Barka N (2016). Defluoridation of groundwater by calcined Mg/Al layered double hydroxide. Emerg. Contam..

[19] Tang H, Wang J, Zhang S, Pang H, Wang X, Chen Z, Yu S (2021). Recent advances in nanoscale zero-valent iron-based materials: Characteristics, environmental remediation and challenges. J. Clean. Prod..

[20] Jarrah NA (2010). Adsorption of Cu (II) and Pb (II) from aqueous solution using Jordanian natural zeolite based on factorial design methodology. Desalin. Water Treat..

[21] Cao M, Liu X, Wang W, Gao M, Yang H (2022). Bifunctional two-dimensional copper–aluminum modified filter paper composite for efficient tetracycline removal: Synergy of adsorption and reusability by degradation. Chemosphere.

[22] Thabede PM, Shooto ND, Naidoo EB (2020). Removal of methylene blue dye and lead ions from aqueous solution using activated carbon from black cumin seeds. S. Afr. J. Chem. Eng..

[23] Farias RSD, Buarque HLDB, Cruz MRD, Cardoso LMF, Gondim TDA, Paulo VRD (2018). Adsorption of congo red dye from aqueous solution onto amino-functionalized silica gel. Engenharia sanitária e ambiental.

[24] Guo X, Yin P, Yang H (2018). Superb adsorption of organic dyes from aqueous solution on hierarchically porous composites constructed by ZnAl-LDH/Al (OH)_3_ nanosheets. Microporous Mesoporous Mater..

[25] Yadav BS, Dasgupta S (2022). Effect of time, pH, and temperature on kinetics for adsorption of methyl orange dye into the modified nitrate intercalated MgAl LDH adsorbent. Inorg. Chem. Commun..

[26] Mochane MJ, Magagula SI, Sefadi JS, Sadiku ER, Mokhena TC (2020). Morphology, thermal stability, and flammability properties of polymer-layered double hydroxide (LDH) nanocomposites: A Review. Crystals.

[27] Xu ZP, Zhang J, Adebajo MO, Zhang H, Zhou C (2011). Catalytic applications of layered double hydroxides and derivatives. Appl. Clay Sci..

[28] Daud M, Kamal MS, Shehzad F, Al-Harthi MA (2016). Graphene/layered double hydroxides nanocomposites: A review of recent progress in synthesis and applications. Carbon.

[29] Kuthati Y, Kankala RK, Lee CH (2015). Layered double hydroxide nanoparticles for biomedical applications: Current status and recent prospects. Appl. Clay Sci..

[30] Sajid M, Jillani SMS, Baig N, Alhooshani K (2022). Layered double hydroxide-modified membranes for water treatment: Recent advances and prospects. Chemosphere.

[31] Rathee G, Awasthi A, Sood D, Tomar R, Tomar V, Chandra R (2019). A new biocompatible ternary Layered Double Hydroxide Adsorbent for ultrafast removal of anionic organic dyes. Sci. Rep..

[32] Rohit RC, Jagadale AD, Shinde SK, Kim DY, Kumbhar VS, Nakayama M (2021). Hierarchical nanosheets of ternary CoNiFe layered double hydroxide for supercapacitors and oxygen evolution reaction. J. Alloy. Compd..

[33] Machrouhi A, Taoufik N, Elhalil A, Tounsadi H, Rais Z, Barka N (2022). Patent Blue V dye adsorption by fresh and calcined Zn/Al LDH: Effect of process parameters and experimental design optimization. J. Compos. Sci..

[34] Darmograi G, Prelot B, Layrac G, Tichit D, Martin-Gassin G, Salles F, Zajac J (2015). Study of adsorption and intercalation of orange-type dyes into Mg–Al layered double hydroxide. J. Phys. Chem. C.

[35] Vishal K, Aruchamy K, Sriram G, Ching YC, Oh TH, Hegde G, Kurkuri M (2023). Engineering a low-cost diatomite with Zn–Mg–Al Layered triple hydroxide (LTH) adsorbents for the effectual removal of Congo red: Studies on batch adsorption, mechanism, high selectivity, and desorption. Colloids Surf. A.

[36] Poudel MB, Kim HJ (2022). Confinement of Zn–Mg–Al-layered double hydroxide and α-Fe_2_O_3_ nanorods on hollow porous carbon nanofibers: A free-standing electrode for solid-state symmetric supercapacitors. Chem. Eng. J..

[37] Zhang J, Lu W, Zhan S, Qiu J, Wang X, Wu Z, Peng H (2021). Adsorption and mechanistic study for humic acid removal by magnetic biochar derived from forestry wastes functionalized with Mg/Al-LDH. Sep. Purif. Technol..

[38] Khatem R, Miguel RO, Bakhti A (2015). Use of synthetic clay for removal of diclofenac anti-inflammatory. Eurasian J. Soil Sci..

[39] Morel-Desrosiers N, Pissón J, Israëli Y, Taviot-Guého C, Besse JP, Morel JP (2003). Intercalation of dicarboxylate anions into a Zn–Al–Cl layered double hydroxide: Microcalorimetric determination of the enthalpies of anion exchange. J. Mater. Chem..

[40] Ogawa M, Asai S (2000). Hydrothermal synthesis of layered double hydroxide–deoxycholate intercalation compounds. Chem. Mater..

[41] Oh JM, Hwang SH, Choy JH (2002). The effect of synthetic conditions on tailoring the size of hydrotalcite particles. Solid State Ionics.

[42] Seida Y, Nakano Y, Nakamura Y (2002). Crystallization of layered double hydroxides by ultrasound and the effect of crystal quality on their surface properties. Clays Clay Miner..

[43] Erickson KL, Bostrom TE, Frost RL (2005). A study of structural memory effects in synthetic hydrotalcites using environmental SEM. Mater. Lett..

[44] Cruz, I. F., Freire, C., Araújo, J. P., Pereira, C., & Pereira, A. M.. Multifunctional ferrite nanoparticles: from current trends toward the future. In *Magnetic Nanostructured Materials* pp. 59–116. (Elsevier, 2018).‏

[45] Sayed H, Mahmoud R, Mohamed HFM, Gaber Y, Shehata N (2022). Co and Ni double substituted Zn–Fe layered double hydroxide as 2D nano-adsorbent for wastewater treatment. Key Eng. Mater..

[46] Wu W, Jiang CZ, Roy VA (2016). Designed synthesis and surface engineering strategies of magnetic iron oxide nanoparticles for biomedical applications. Nanoscale.

[47] Abdel-Hady EE, Mahmoud R, Hafez SH, Mohamed HFM (2022). Hierarchical ternary ZnCoFe layered double hydroxide as efficient adsorbent and catalyst for methanol electrooxidation. J. Market. Res..

[48] Gao M, Wang W, Cao M, Yang H, Li Y (2020). Hierarchical hollow manganese–magnesium–aluminum ternary metal oxide for fluoride elimination. Environ. Res..

[49] Zaher A, Taha M, Farghali AA, Mahmoud RK (2020). Zn/Fe LDH as a clay-like adsorbent for the removal of oxytetracycline from water: Combining experimental results and molecular simulations to understand the removal mechanism. Environ. Sci. Pollut. Res..

[50] Nazir MA, Bashir MA, Najam T, Javed MS, Suleman S, Hussain S, ur Rehman A (2021). Combining structurally ordered intermetallic nodes: Kinetic and isothermal studies for removal of malachite green and methyl orange with mechanistic aspects. Microchem. J..

[51] Abo El-Reesh GY, Farghali AA, Taha M, Mahmoud RK (2020). Novel synthesis of Ni/Fe layered double hydroxides using urea and glycerol and their enhanced adsorption behavior for Cr (VI) removal. Sci. Rep..

[52] Huang L, Wang L, Wang C, Tao X (2022). Effect of intercalation of flocculant on adsorption properties of ZnMgAl-LDHs. Inorg. Chem. Commun..

[53] Tao X, Liu D, Song J, Ye Q, Xu D (2017). Plasma modification of ZnMgAl-LDHs for adsorption property improvement. J. Taiwan Inst. Chem. Eng..

[54] Bernardo MP, Ribeiro C (2018). [Mg–Al]-LDH and [Zn–Al]-LDH as matrices for removal of high loadings of phosphate. Mater. Res..

[55] Bakr AA, Eshaq G, Rabie AM, Mady AH, ElMetwally AE (2016). Copper ions removal from aqueous solutions by novel Ca–Al–Zn layered double hydroxides. Desalin. Water Treat..

[56] Eshaq G, Rabie AM, Bakr AA, Mady AH, ElMetwally AE (2016). Cr (VI) adsorption from aqueous solutions onto Mg–Zn–Al LDH and its corresponding oxide. Desalin. Water Treat..

[57] Wang J, Kang D, Yu X, Ge M, Chen Y (2015). Synthesis and characterization of Mg–Fe–La trimetal composite as an adsorbent for fluoride removal. Chem. Eng. J..

[58] Ji H, Wu W, Li F, Yu X, Fu J, Jia L (2017). Enhanced adsorption of bromate from aqueous solutions on ordered mesoporous Mg–Al layered double hydroxides (LDHs). J. Hazard. Mater..

[59] Wen T, Wu X, Tan X, Wang X, Xu A (2013). One-pot synthesis of water-swellable Mg–Al layered double hydroxides and graphene oxide nanocomposites for efficient removal of As (V) from aqueous solutions. ACS Appl. Mater. Interfaces.

[60] Kuśtrowski P, Rafalska-Łasocha A, Majda D, Tomaszewska D, Dziembaj R (2001). Preparation and characterization of new Mg–Al–Fe oxide catalyst precursors for dehydrogenation of ethylbenzene in the presence of carbon dioxide. Solid State Ionics.

[61] Ghorbani-Choghamarani A, Tahmasbi B (2016). The first report on the preparation of boehmite silica sulfuric acid and its applications in some multicomponent organic reactions. New J. Chem..

[62] Zheng YM, Li N, Zhang WD (2012). Preparation of nanostructured microspheres of Zn–Mg–Al layered double hydroxides with high adsorption property. Colloids Surf. A.

[63] Kristian, R. The influence of surface roughness on protein adsorption. *Thesis* (2006).‏

[64] Huang YW, Gupta VK (2004). A SPR and AFM study of the effect of surface heterogeneity on adsorption of proteins. J. Chem. Phys..

[65] https://www.digitalsurf.com/free-trial/.

[66] Ţălu Ş, Shcherbinin DP, Konshina EA, Gladskikh IA (2021). Stereometric and fractal analysis of granulated silver films used in thin-film hybrid structures. J. Microsc..

[67] Najjar D, Bigerelle M, Migaud H, Iost A (2005). Identification of scratch mechanisms on a retrieved metallic femoral head. Wear.

[68] Ţălu Ş, Janus K, Stach S (2017). Nanoscale patterns in carbon–nickel nanocomposite thin films investigated by AFM and stereometric analysis. Int. J. Mater..

[69] Kumar BR, Rao TS (2012). AFM studies on surface morphology, topography and texture of nanostructured zinc aluminum oxide thin films. Dig. J. Nanomater. Biostruct..

[70] Dalla Nora FB, Lima VV, Oliveira ML, Hosseini-Bandegharaei A, de Lima Burgo TA, Meili L, Dotto GL (2020). Adsorptive potential of Zn–Al and Mg–Fe layered double hydroxides for the removal of 2–nitrophenol from aqueous solutions. J. Environ. Chem. Eng..

[71] Mebrahtu G, Zerabruk S (2011). Concentration and health implication of heavy metals in drinking water from urban areas of Tigray region, Northern Ethiopia. Momona Ethiopian J. Sci..

[72] Mohod CV, Dhote J (2013). Review of heavy metals in drinking water and their effect on human health. Int. J. Innov. Res. Sci. Eng. Technol..

[73] Ali SHB (2012). Experimental Analysis on Quality of Drinking Water in State of Perak.

[74] Bharali D, Deka RC (2017). Preferential adsorption of various anionic and cationic dyes from aqueous solution over ternary CuMgAl layered double hydroxide. Colloids Surf. A.

[75] Gao M, Wang W, Cao M, Yang H, Li Y (2020). Constructing hydrangea-like hierarchical zinc-zirconium oxide microspheres for accelerating fluoride elimination. J. Mol. Liq..

[76] Heidarinejad Z, Rahmanian O, Fazlzadeh M, Heidari M (2018). Enhancement of methylene blue adsorption onto activated carbon prepared from Date Press Cake by low frequency ultrasound. J. Mol. Liq..

[77] Gao M, Wang W, Yang H, Ye BC (2020). Efficient removal of fluoride from aqueous solutions using 3D flower-like hierarchical zinc-magnesium-aluminum ternary oxide microspheres. Chem. Eng. J..

[78] Rashed SH, Abd-Elhamid AI, Abdalkarim SYH, El-Sayed RH, El-Bardan AA, Soliman HM, Nayl AA (2022). Preparation and characterization of layered-double hydroxides decorated on graphene oxide for dye removal from aqueous solution. J. Market. Res..

[79] Lu L, Li J, Ng DH, Yang P, Song P, Zuo M (2017). Synthesis of novel hierarchically porous Fe_3_O_4_@ MgAl–LDH magnetic microspheres and its superb adsorption properties of dye from water. J. Ind. Eng. Chem..

[80] Zubair M, Jarrah N, Manzar MS, Al-Harthi M, Daud M, Mu’azu ND, Haladu SA (2017). Adsorption of eriochrome black T from aqueous phase on MgAl-, CoAl-and NiFe-calcined layered double hydroxides: Kinetic, equilibrium and thermodynamic studies. J. Mol. Liq..

[81] Bulut Y, Aydın HA (2006). kinetics and thermodynamics study of methylene blue adsorption on wheat shells. Desalination.

[82] Volesky B, Weber J, Park JM (2003). Continuous-flow metal biosorption in a regenerable Sargassum column. Water Res..

[83] Kaveeshwar AR, Ponnusamy SK, Revellame ED, Gang DD, Zappi ME, Subramaniam R (2018). Pecan shell based activated carbon for removal of iron (II) from fracking wastewater: Adsorption kinetics, isotherm and thermodynamic studies. Process Saf. Environ. Prot..

[84] Ai L, Zhang C, Meng L (2011). Adsorption of methyl orange from aqueous solution on hydrothermal synthesized Mg–Al layered double hydroxide. J. Chem. Eng. Data.

[85] Qu J, Tian X, Jiang Z, Cao B, Akindolie MS, Hu Q, Zhang Y (2020). Multi-component adsorption of Pb (II), Cd (II) and Ni (II) onto microwave-functionalized cellulose: Kinetics, isotherms, thermodynamics, mechanisms and application for electroplating wastewater purification. J. Hazard. Mater..

[86] Shu D, Feng F, Han H, Ma Z (2017). Prominent adsorption performance of amino-functionalized ultra-light graphene aerogel for methyl orange and amaranth. Chem. Eng. J..

[87] Nebaghe KC, El Boundati Y, Ziat K, Naji A, Rghioui L, Saidi M (2016). Comparison of linear and non-linear method for determination of optimum equilibrium isotherm for adsorption of copper (II) onto treated Martil sand. Fluid Phase Equilib..

[88] Zhang JZ (2011). Avoiding spurious correlation in analysis of chemical kinetic data. Chem. Commun..

[89] Zhou J, Lü QF, Luo JJ (2017). Efficient removal of organic dyes from aqueous solution by rapid adsorption onto polypyrrole—Based composites. J. Clean. Prod..

[90] Ghosh A, Das G (2020). Green synthesis of Sn (II)-BDC MOF: Preferential and efficient adsorption of anionic dyes. Microporous Mesoporous Mater..

[91] Jiang H, Yang Y, Lin Z, Zhao B, Wang J, Xie J, Zhang A (2020). Preparation of a novel bio-adsorbent of sodium alginate grafted polyacrylamide/graphene oxide hydrogel for the adsorption of heavy metal ion. Sci. Total Environ..

[92] Hlekelele L, Nomadolo NE, Setshedi KZ, Mofokeng LE, Chetty A, Chauke VP (2019). Synthesis and characterization of polyaniline, polypyrrole and zero-valent iron-based materials for the adsorptive and oxidative removal of bisphenol-A from aqueous solution. RSC Adv..

[93] Değermenci GD, Değermenci N, Ayvaoğlu V, Durmaz E, Çakır D, Akan E (2019). Adsorption of reactive dyes on lignocellulosic waste; characterization, equilibrium, kinetic and thermodynamic studies. J. Clean. Prod..

[94] Grover A, Mohiuddin I, Malik AK, Aulakh JS, Vikrant K, Kim KH, Brown RJ (2022). Magnesium/aluminum layered double hydroxides intercalated with starch for effective adsorptive removal of anionic dyes. J. Hazard. Mater..

[95] Wan Y, Liu ZY, Song P, Zhang XQ, Song JC, Fu YJ, Shi CY (2019). Ionic liquid groups modified 3D porous cellulose microspheres for selective adsorption of AO_7_ dye. J. Clean. Product..

[96] Mu'azu ND, Jarrah N, Kazeem TS, Zubair M, Al-Harthi M (2018). Bentonite-layered double hydroxide composite for enhanced aqueous adsorption of Eriochrome Black T. Appl. Clay Sci..

[97] Kazeem TS, Zubair M, Daud M, Mu’azu ND, Al-Harthi MA (2019). Graphene/ternary layered double hydroxide composites: Efficient removal of anionic dye from aqueous phase. Korean J. Chem. Eng..

[98] Mashkoor F, Nasar A (2020). Magnetized Tectona grandis sawdust as a novel adsorbent: Preparation, characterization, and utilization for the removal of methylene blue from aqueous solution. Cellulose.

[99] Mazloom F, Masjedi-Arani M, Ghiyasiyan-Arani M, Salavati-Niasari M (2016). Novel sodium dodecyl sulfate-assisted synthesis of Zn_3_V_2_O_8_ nanostructures via a simple route. J. Mol. Liq..

[100] Wang W, Gao M, Cao M, Liu X, Yang H, Li Y (2021). A series of novel carbohydrate-based carbon adsorbents were synthesized by self-propagating combustion for tetracycline removal. Biores. Technol..

[101] Zhang Q, Cheng Y, Fang C, Chen J, Chen H, Li H, Yao Y (2020). Facile synthesis of porous carbon/Fe_3_O_4_ composites derived from waste cellulose acetate by one-step carbothermal method as a recyclable adsorbent for dyes. J. Market. Res..

[102] He S, Liu X, Yan P, Wang A, Su J, Su X (2019). Preparation of gemini surfactant/graphene oxide composites and their superior performance for Congo red adsorption. RSC Adv..

[103] Leng L, Yuan X, Zeng G, Shao J, Chen X, Wu Z, Peng X (2015). Surface characterization of rice husk bio-char produced by liquefaction and application for cationic dye (Malachite green) adsorption. Fuel.

[104] Zhu X, Liu Y, Zhou C, Luo G, Zhang S, Chen J (2014). A novel porous carbon derived from hydrothermal carbon for efficient adsorption of tetracycline. Carbon.

